# 2003. Igniting the Spark: An Exploratory Qualitative Study of Medical Student Preclinical Infectious Diseases Learning

**DOI:** 10.1093/ofid/ofad500.130

**Published:** 2023-11-27

**Authors:** Elizabeth Scruggs-Wodkowski, Emily Abdoler

**Affiliations:** University of Michigan, Ann Arbor, MI; University of Michigan, Ann Arbor, MI

## Abstract

**Background:**

Major educational goals of Infectious Diseases (ID) are to recruit more trainees into ID and promote good antimicrobial decision-making. National survey research (Bonura et al, 2016) revealed that ID curricula that deemphasize memorization are associated with greater odds of trainees entering the field of ID. As ID electives are not required during medical school, preclinical ID courses could represent the last dedicated opportunity for students to gain exposure to the field and acquire key clinical skills for antimicrobial selection. Yet little is known about how students experience preclinical ID courses. We aimed to explore how students experience and engage with these courses to better understand how best to augment their learning and interest in ID.

**Methods:**

We conducted individual interviews with a purposeful sample of medical students who took the preclinical ID course during three curricular years (2019-2021). Our semi-structured interviews explored students' challenges in learning ID, as well as the motivations, learning principles, and strategies guiding that learning. We generated a codebook through an iterative, inductive process using Dedoose to code interviews and facilitate analysis. We confirmed and refined major themes over 19 interviews.

**Results:**

Students face unique challenges learning ID but are motivated to succeed by both intrinsic and extrinsic factors (Table 1). We identified six core learning principles (*Establishing Relevance, Organized Study, Relating Ideas, Multi-Modal Learning, Socially Constructed Learning,* and *Active Engagement with Material*) that support student learning, deployed through four main strategies (*Peer Advice, Writing it Out, Talking it Out,* and *Use of External Resources*) (Table 2; Figure 1).

**Table 1.**
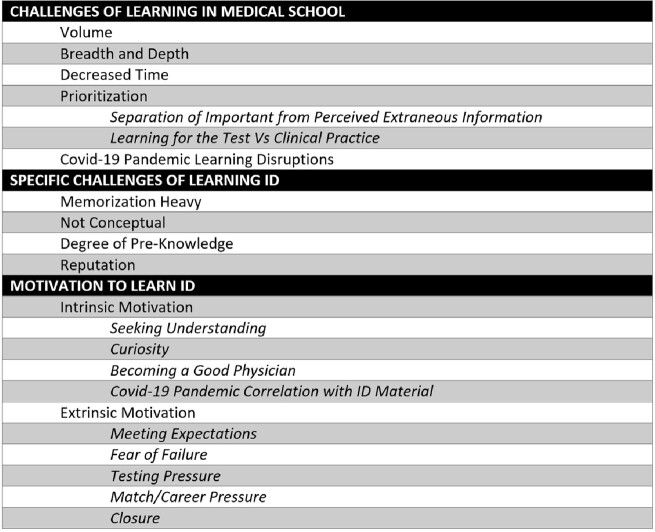
Challenges and Motivations in Learning Infectious Diseases (ID)

**Table 2.**
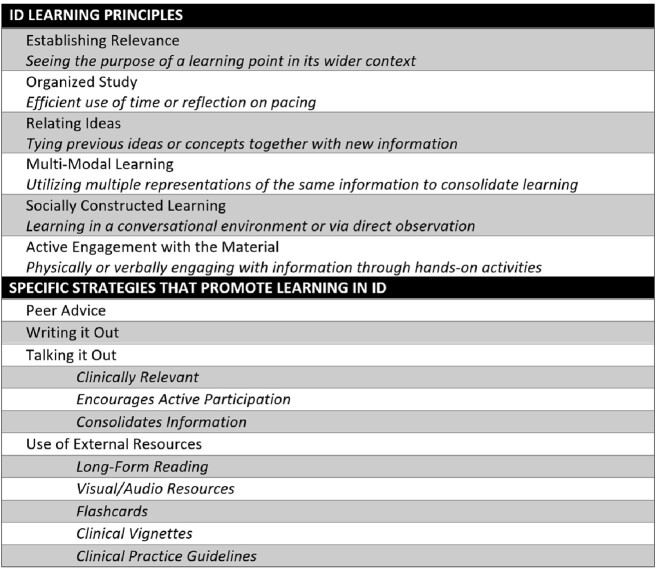
Infectious Diseases (ID) Learning Principles and Strategies

**Figure 1.**
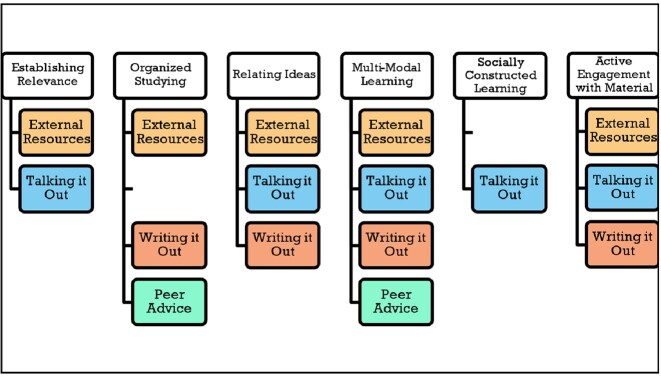
Infectious Diseases (ID) Learning Principles with Supportive Strategies

**Conclusion:**

Our results build on prior research indicating that students learning ID benefit from incorporation of educational principles and strategies that move beyond rote memorization to encourage active engagement and self-reflection. We found that students preference deep, strategic learning methods propelled by intrinsic motivation, despite external pressures. Our findings have implications for optimizing ID education and ultimately increasing interest in the field.

**Disclosures:**

**All Authors**: No reported disclosures

